# Disability benefits protect against lost income for South Africans living with Schizophrenia

**DOI:** 10.1007/s00127-023-02577-y

**Published:** 2023-10-17

**Authors:** Olivia Wootton, Aisha King, Kayley Moy, Dan J. Stein, Ezra S. Susser

**Affiliations:** 1 Department of Psychiatry and Neuroscience Institute, University of Cape Town, Cape Town, South Africa; 2 Graduate School of Public Health and Health Policy, City University of New York, New York, NY USA; 3 Department of Global Public Health, New York University, New York City, USA; 4 SAMRC Unit on Risk and Resilience in Mental Disorders, Cape Town, South Africa; 5 Department of Epidemiology, Mailman School of Public Health, Columbia University, New York, USA; 6 New York State Psychiatric Institute, New York, NY USA

**Keywords:** Schizophrenia, South Africa, Social security disability, Socioeconomic status (SES)

## Abstract

Little is known about the economic impact of disability grants for people living with schizophrenia in low- and middle- income countries. In this brief report, we show that receipt of disability benefits is significantly associated (*β* = 0.105, *p* < 0.0001) with increased household and personal wealth in large sample of people living with schizophrenia in South Africa (*n* = 1154). This study provides further support for the use of disability grants as a mechanism to protect people living with schizophrenia and their families against the economic costs associated with schizophrenia.

## Introduction

Schizophrenia is among the leading causes of disability globally, and has been under-researched in low resource settings [1]. People living with chronic schizophrenia (PLS) often suffer extreme economic hardships such as being unemployed and living in deep poverty for long periods of time [2–4]. The families of PLS may also suffer financially from the costs of intensive care, and may face significant emotional burdens and psychosocial challenges brought on by stigmatization, anxiety, and resentment [5–7]. These hardships are especially salient in lower-resource settings like South Africa, where mental health services are difficult to access [8–10] and where economic hardship is common in the general population.

Social services such as disability grants have been proposed as one mechanism to increase independence and quality of life among PLS. In South Africa, PLS may qualify for a disability grant if they meet the legal requirements (e.g. South African citizenship, aged 18–59 years), satisfy a means test, are not in the care of the state, and their illness impairs their ability to work. The impact of disability grants has been extensively studied in high-income countries [11–13] and evidence suggests that many PLS rely on disability grants to alleviate the economic pressure brought on by unemployment [14, 15]. At the same time, some research suggests that receipt of disability grants could have unintended adverse consequences, including reduced motivation to seek employment and worsening of negative symptoms [12, 16, 17]. However, there has been little research on the impact of disability grants among PLS in lower-resource settings, where unemployment rates, poverty, and inequalities are even more pronounced [18, 19].

This report from South Africa examines whether disability grants are associated with a reduction of financial hardship and poverty in PLS. We compare predictors of household wealth, including receipt of a disability grant, in a group of PLS. To our knowledge, this is the first large study of the potential benefits of disability grants for PLS in a lower-resource setting.

## Methods

Data were collected during the Genomics of Schizophrenia in the South African Xhosa people (SAX) study [20]. SAX was a case–control study that aimed to characterize the genetic architecture of schizophrenia in the Xhosa population, one of the largest ethnic groups in South Africa. A total of 2849 participants who self-identified as Xhosa were recruited from health clinics and psychiatric hospitals in the Western and Eastern Capes of South Africa between 2013 and 2018. Enrollment procedures are described in previous publications [20–22]. The current report includes cases (*n* = 1154), defined as individuals with the capacity to provide informed consent and a diagnosis of schizophrenia or schizoaffective disorder for at least 2 years prior to enrolment. Demographic data, including information on assets, income, and access to social assistance, was collected. All participants completed the Structured Clinical Interview for DSM-IV Diagnoses [23]. This study complies with the Declaration of Helsinki and was approved by the University of Cape Town Human Research Ethics Committee (reference number—049/2013).

For this secondary data analysis, cases were divided into two groups: those receiving a disability grant (*n* = 655; 57%) and those not receiving a disability grant (*n* = 499; 43%). Differences in demographic characteristics were assessed using Chi square tests.

To adequately capture economic status in this setting where resource sharing in households is common [24], household and personal wealth were assessed using a 12-item asset index, based on the index used in the South African Stress and Health Study [25]. The asset index included household amenities (electricity, running water, flushing toilets), other household resources (telephone, bicycle, motor vehicle, electric stove, kitchen sink), and participation in other financial activities (shopping at supermarkets, use of financial services, retail store accounts, lay-by payments). The asset index demonstrated acceptable inter-rater reliability (*α* = 0.69). Multiple linear regression was used to assess the relationship between asset index score and the following independent variables: receipt of a disability grant, sex, age, educational attainment, employment status, marital status, number of household members, and location (rural/urban). All statistical analyses were performed using IBM SPSS version 28.0.1.0.

## Results

### Sample characteristics

Of the 1,154 cases (13.7% female; mean age 36.3 years), 57% (*n* = 655) were currently receiving a disability grant. A majority of cases reported unemployment (94%), did not complete secondary education (82%), were unmarried (91%), and resided in an urban setting (89%). PLS who were receiving a disability grant were significantly older (mean age 38.4 v. 35.6, *p* < 0.001) and reported higher rates of unemployment (96% v. *91*%, *p* < 0.001) than PLS who were not receiving disability grants. Level of education, sex, household urbanicity, province of residence, marital status and number of household members did not differ significantly between the two groups.

### Predictors of household wealth/assets

In the full model, higher number of household assets was significantly associated with receipt of a disability grant, higher level of education, current employment, higher number of household members, and living in an urban area (Table 1**)**. Among these, level of education (*β* = 0.267, *p* < 0.0001) and living in an urban setting (*β* = 0.216, *p* < 0.0001), had the largest effect on number of household assets. Sex, age, and marital status did not have a significant relationship with the asset index.

**Table 1:** Linear regression of selected predictor variables and household wealth among people with Schizophrenia

Predictor variable	*B*	95% CI	*β*	*p*
Receipt of disability grant	0.407	[0.167, 0.648]	0.105	< 0.001***
Sex	0.015	[− 0.324, 0.355]	0.003	0.930
Age	− 0.032	[− 0.180, 0.118]	− 0.013	0.685
Level of education	0.595	[0.457, 0.734]	0.267	< 0.001***
Employment	0.793	[0.298, 1.288]	0.099	0.002**
Marital status	− 0.243	[− 0.656, 0.170]	− 0.036	0.248
Number of Household members	0.647	[0.426, 0.868]	0.177	< 0.001***
Location	1.344	[0.967, 1.721]	0.216	< 0.001***
Constant = 1.447, *F*(8,868) = 24.392, *p* < 0.001***, adj. *R*^2^ = 0.176				

*B* unstandardized regression coefficient*, β* standardized regression coefficient, *CI* confidence interval

*p < 0.05, **p < 0.01, ***p < 0.001

## Discussion

Our results suggest that receipt of a disability grant is an important determinant of household wealth amongst PLS in South Africa. Additionally, we identify other significant predictors of household wealth amongst PLS, emphasizing the relative importance of level of education and area of residence in determining economic status in this setting.

The percentage of participants that reported receipt of disability grant (57%) was higher than the percentage reported in previous studies of mental healthcare users in South Africa [26, 27]. Unlike previous research, this study only included participants with a diagnosis of schizophrenia, who may be more likely to qualify for receipt of disability benefits than other mental health care users. During the study period, when the unemployment rate in South Africa ranged from 24.5 to 26.9% [18], most participants (94%) in this study reported being unemployed. One of the frequently cited disadvantages of disability benefits is that they discourage recipients from seeking employment [12, 16]. In South Africa, PLS may still qualify for a disability grant if they earn below a certain amount (range: R47,400–R78,120 for the study period). Our results show that PLS who were receiving disability benefits were less likely to be employed than PLS not receiving disability benefits. However, we cannot assess directionality based on the present data, therefore it is possible that PLS who were employed were less likely to be referred for a disability grant in the first place, as this is up to clinicians’ discretion. Research has found that even eligible individuals may have difficulties accessing disability grants for a variety of reasons [28]. Regardless, the unemployment rate amongst both groups of PLS was substantially higher than the national average. This finding provides further evidence of the impairment in occupational functioning that is typical of schizophrenia and the potential economic costs of the disorder [1, 2, 29]. In this context, where national unemployment rates remain high and supported employment opportunities are scarce [30], we suggest that withholding disability benefits from PLS to encourage employment may be harmful.

We show that disability grants were predictive of household wealth in our sample, demonstrating that disability benefits may help protect PLS and their families against the economic costs of the disorder. Regarding other predictors of wealth, we found that the most significant predictors of higher household assets were level of education and living in an urban setting. This finding is consistent with the extensive body of evidence that supports a positive association between educational attainment and improved economic outcomes, including earnings and employment opportunities [31–33]. Our results add to existing evidence by demonstrating this association holds among people living with schizophrenia in a LMIC. The finding that living in an urban area is predictive of household wealth is consistent with prior studies that show higher poverty rates among rural compared to urban areas in South Africa. The history of land dispossession and migratory labour patterns during periods of colonialism and apartheid have contributed to a lack of resources and employment opportunities among historically marginalized groups in the rural areas, perpetuating rural poverty [19, 34]. Lastly, we found that number of household members was predictive of household wealth, which most likely reflects pooling of resources by household members.

There are some limitations to this study. First, the data were cross-sectional, which precludes causal interpretation of the significant association between sociodemographic factors and household wealth. Second, this was a secondary data analysis and only one ethnic group was included in the primary study. Thus, our ability to fully explore the benefits and adverse effects associated with disability grants in South Africa was limited by the available data. Third, since the study’s completion in 2018, the socioeconomic climate in South Africa has changed. While we hypothesize that most of our findings remain relevant, further research is required to confirm current predictors of wealth. Lastly, migration between rural and urban areas in South Africa is common [35] and the association between living in an urban setting and receipt of a disability grant should be interpreted with caution.

To the best of our knowledge, this is the first large scale exploration of the determinants of household and personal wealth amongst PLS in two economically diverse regions within South Africa. The results of this study emphasize the important role of disability benefits in protecting against the consequences of lost income associated with schizophrenia.

## Data Availability

The data that support the findings of this study are available from The National Institute of Mental Health Data Archive. Restrictions apply to the availability of these data. Data are available upon request from https://nda.nih.gov/edit_collection.html?id=3781.
